# Disorientation as a delirium feature in non-intubated patients: development and evaluation of diagnostic accuracy of the ‘Confusion Assessment Method for Intermediate Care Unit’ (CAM-IMC) - a prospective cohort study

**DOI:** 10.1186/s12871-024-02849-3

**Published:** 2024-12-13

**Authors:** L. P. Beyer, L. von zur Gathen, B. El Rayah, O. Dewald, T. Zieschang, A. Diers, E. Wesley Ely, U. Guenther

**Affiliations:** 1 Fakultät VI – Medizin und Gesundheitswissenschaften, Carl von Ossietzky Universität, Oldenburg, Germany; 2 Universitätsklinik für Anästhesiologie/Intensivmedizin/Notfallmedizin/Schmerztherapie, Klinikum Oldenburg AöR, Universitätsmedizin Oldenburg, Oldenburg, Germany; 3 Universitätsklinik für Herzchirurgie, Universitätsklinikum Erlangen, Erlangen, Germany; 4 Critical Illness, Brain Dysfunction, and Survivorship (CIBS) Center, Vanderbilt University Medical Center, Nashville, USA; 5 Department of Medicine, Division of Pulmonary and Critical Care Medicine, Vanderbilt University School of Medicine, Nashville, USA; 6 Geriatric Research Education Clinical Center (GRECC), Department of Veterans Affairs, Tennessee Valley Healthcare System, Nashville, USA; 7 Universitätsklinik für Geriatrie, Klinikum Oldenburg AöR, Oldenburg, Germany; 8 Universitätsklinik für Intensivmedizin, Klinikum Oldenburg AöR, Rahel-Straus-Straße 10, 26133 Oldenburg, Germany

**Keywords:** CAM-IMC, CAM-ICU, POD, Delirium, Non-intubated, Disorientation

## Abstract

**Background:**

Disorientation is an early indicator of developing postoperative delirium (POD), which is associated with increased mortality and cognitive decline. The well-established “Confusion-Assessment-Method-for-Intensive-Care-Unit” (CAM-ICU) for diagnosing POD in intubated patients cannot make use of the feature ‘disorientation’, as this requires verbal communication. Other tools such as the 4AT test for disorientation but are not established in ICU settings. We therefore combined test-variables of the CAM-ICU (level of consciousness, fluctuating mental status and inattention) with verbal testing for disorientation to develop and enhance diagnostic accuracy of the “Confusion Assessment Method for Intermediate Care Unit” (CAM-IMC). In the present study we describe the development and the evaluation of the diagnostic accuracy of the CAM-IMC.

**Methods:**

We conducted a prospective cohort-study to develop and evaluate the diagnostic accuracy of the CAM-IMC and disorientation for diagnosing POD in non-intubated patients undergoing elective cardiac surgery. All patients were eligible during data collection period. Exclusion criteria were preexisting brain-organic disease, age < 50 years, preoperative intubation, and insufficient language skills. Patients were assessed for POD using the CAM-IMC as the index-test by two independent examiners over three postoperative days. Reference-testing was conducted by experienced reference-raters. The primary outcome was the diagnostic test-performance.

**Results:**

Among 178 eligible patients, 624 paired observations were completed with 155 patients. Of these, 9% experienced POD. Sensitivity and specificity were 0.96 (CI-95%: 0.87-1.00) and 0.94 (CI-95%: 0.92–0.96), respectively. Area-Under-the-Receiver-Operating-Characteristic-Curve (AUROC; equivalent to c-statistic) for CAM-IMC with a cut-off at three points was 0.95 (CI-95%: 0.93–0.98). The interrater reliability was 0.80 (CI-95%: 0.69–0.91).

**Conclusion:**

The CAM-IMC demonstrates excellent test performance for diagnosing POD in non-intubated patients by combining features of the CAM-ICU with ‘disorientation’. Given an aging community with an increasing delirium risk, the CAM-IMC provides a highly structured assessment tool for POD. It enables early and accurate detection of delirium, which is critical for timely intervention and improved patient outcomes. The CAM-IMC appears to be a useful tool to be implemented in units for not-intubated patients and seems to be the perfect match where the CAM-ICU is already in use for monitoring POD.

**Trial registration:**

DRKS00026980 (German registry of clinical studies).

## Introduction

Disorientation is an early sign of developing postoperative delirium (POD) [1]. POD is reported to occur in about 1/3 of ICU-patients and is characterized by acute onset or fluctuating course of mental status, inattention, and a varying degree of other cognitive dysfunctions [2, 3]. Also, disturbances of emotion, behavior and the sleep-wake cycle may be present [2]. On an individual basis, though symptoms can be transitory, POD may be associated with prolonged hospitalization, delayed rehabilitation, further cognitive decline, increase in risk of mortality and posttraumatic stress disorder [4–11]. The economic effects are substantial, since POD was shown to be associated with additional healthcare costs of around $45,000 per patient, making it a large-scale public health issue [12].

Risk factors include non-modifiable conditions including advanced age, impaired pre-existing cognition, significant comorbidities, and potentially modifiable (precipitating) factors such as deep sedation, untreated trauma, pharmacological interactions, and new-onset infections [13, 14]. Particularly the latter require urgent treatment and make quick diagnosis of POD mandatory. Early recognition of POD features is key to delirium management, as there are several modifiable risk factors, such as new-onset infections, pain, inappropriate drug use and pharmacological interactions [13, 14]. Since the predominant manifestation is hypoactive delirium, exhibiting no or reduced motor activity, it often goes undiagnosed [15–18].

The Confusion-Assessment-Method for Intensive Care Unit (CAM-ICU), which originally derived from the Confusion Assessment Method (CAM), was developed for monitoring of POD in intubated ICU-patients [19, 20]. It omits the feature ‘disorientation’, as this requires a more differentiated verbal response than ‘yes’ and ‘no’, or nodding ‘yes’ and shaking your head ‘no’. Disorientation should not be missed as a frequent symptom and early indicator of POD as stated above. Screening for disorientation has been shown to provide high sensitivity (96.6%) in the diagnosis of POD [21].

A substantial proportion of critically ill patients are nowadays not orally intubated and thus amenable to verbal assessments [22, 23]. We therefore sought to combine the highly operationalized CAM-ICU features with ‘disorientation’ to develop the ‘Confusion Assessment Method for Intermediate Care Unit’ (CAM-IMC). An Intermediate-Care-Unit (IMC) is a unit for patients without need for invasive ventilation but in need for non-invasive organ-support or close monitoring. In alignment with the nomenclature of the CAM-ICU for intubated patients we named the assessment-tool CAM-IMC. A retrospective analysis for disorientation in assessment of POD indicated that the CAM-IMC could provide a sensitive bedside diagnostic test to detect POD in non-intubated patients [24].

This prospective cohort study was conducted to develop and evaluate the test performance of both ‘disorientation’ and the CAM-IMC in diagnosing POD in non-intubated cardiac-surgery patients compared to experienced reference-raters.

## Methods

### Study design

This study is a prospective cohort study conducted at a tertiary care hospital in Oldenburg, Germany. It was performed in line with the principles of the Declaration of Helsinki. Ethics approval was obtained through the University of Oldenburg medical ethics committee (2020-020). This manuscript follows the STARD reporting standard for studies of diagnostic accuracy [25].

### Patients

All elective cardiac surgery patients were eligible for the study protocol during data collection time and were included consecutively. Exclusion criteria were preexisting brain-organic disease (e.g. dementia or acute stroke), age younger than 50 years, preoperative intubation, and insufficient German language skills. The cut-off of 50 years was chosen to obtain a higher proportion of patients with POD. Patients were excluded if insufficient language skills prevented effective communication. Written consent to participation was obtained prior to data collection and testing.

### Data collection

Data was collected from October 2021 through February 2022. All patients were recruited and assessed prior to their scheduled surgery by two medical students. Preoperative cognition status was tested using the Mini-Cog-Test, along with assessments of all CAM-IMC features including tests of inattention and disorientation [26]. Patient characteristics, including date of birth, age, sex, weight, height, concomitant diseases, planned type of surgery and laboratory parameters (blood count, CRP, creatinine, AST, ALT, cholinesterase) were recorded as part of the clinical routine. After surgery, patients were examined for delirium on the first to third postoperative day.

Study data were collected and managed using REDCap electronic data capture tools hosted at Klinikum Oldenburg [27, 28]. REDCap (Research Electronic Data Capture) is a secure, web-based software platform designed to support data capture for research studies, providing (1) an intuitive interface for validated data capture; (2) audit trails for tracking data manipulation and export procedures; (3) automated export procedures for seamless data downloads to common statistical packages; and (4) procedures for data integration and interoperability with external sources. Duplicate data entry was performed for quality assurance.

### Assessment of delirium

**Fig. 1:**
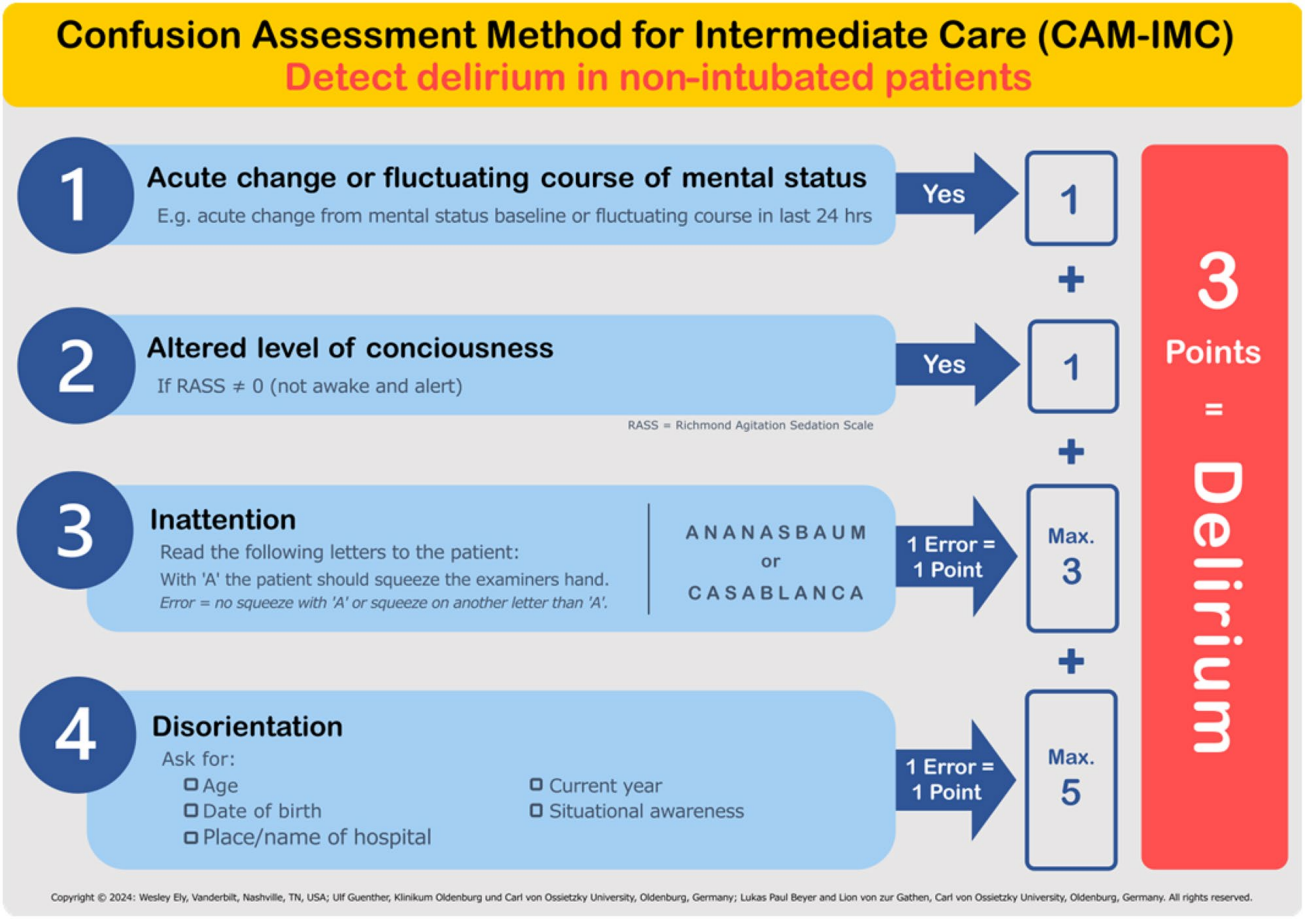
‘Confusion Assessment Method for Intermediate Care Unit’ (CAM-IMC) diagnostic flow chart

Figure 1 illustrates the diagnostic workup using CAM-IMC as the index test. A point-based scoring system was introduced in CAM-IMC, allowing for a modified weighting of each element in the diagnosis of POD. The elements “Acute change or fluctuating course of mental status” and “Altered level of consciousness” are dichotomous, allowing for the assignment of one point for an abnormal test result. Inattention was assessed by having the patient read aloud a ten-letter word (ANANASBAUM or CASABLANCA) and squeeze the examiner’s hand each time they heard the letter “A.” Consistent with the CAM-ICU, a maximum of three errors were permitted, with one point deducted for each mistake. Disorientation was newly introduced and evaluated across five different dimensions. Based on the abbreviated-mental-test-4 (AMT4) which was also the base for the 4AT we chose the four dimensions age, date of birth, place and year [21, 29, 30]. The fifth dimension we added was ‘situational awareness’, as during a prior study by Guenther et al., it was discovered a useful addition [24]. Interestingly, one of the earliest reports on delirium (Levin, 1956) described it as a useful diagnostic criterion, by “asking the patient how to get from one place to another” [1]. In this study we asked about their way they took from onset of symptoms to our hospital. Each error in these dimensions resulted in one point, with a maximum of five points possible.

Postoperative day one through three index testing was obtained by two independent examiners who engaged the patients on that day. These examiners were both residents and nursing staff. For reference rating, patients were assessed for POD by an experienced reference-rater once every day. The reference-raters used DSM-5 criteria, Richmond Agitation-Sedation Scale (RASS), CAM-ICU and features of disorientation for diagnosing POD. The reference rater was allowed to make his diagnosis based on all the diagnostic criteria and his professional experience. The reference raters were experienced senior consultants in intensive care (BER, UG) and geriatrics (TZ). Clinical information, e.g. nurses’ notes and electronic medical records, was available to all examiners. All examination results were blinded in a sealed box. No examiner had knowledge about other index test results or preoperative cognitive testing. The examinations were carried out alongside the morning ward round without no more than 60 min in between examinations.

### Statistical analysis

Analysis was performed using IBM SPSS Statistics 27 and R Statistical language (version 4.2.2; R Core Team, 2022). A priori, a sample size calculation based on an estimated 30% prevalence of POD, indicated a minimum of 156 patients required to provide sufficient diagnostic value calculation. A plausibility check of the data was performed prior to analysis. Missing values were either imputed based on chart review or lead to exclusion and were not used for statistical analysis.

The primary outcome was determination of sensitivity, specificity, positive and negative predictive value, positive and negative likelihood-ratio of the CAM-IMC and disorientation. These values were calculated using 2 × 2 tables. In addition, the Area Under the ROC Curve (AUROC; equivalent to c-statistic) was calculated. A receiver operating characteristics (ROC) curves were plotted to compare the performance of the CAM-IMC and its features inattention, altered level of consciousness, acute change, or fluctuation course of mental status and especially disorientation. To determine the optimal cut-off value of the CAM-IMC the Youden-Index was calculated. Confidence intervals were calculated using Clopper-Pearson-method and DeLong-method. Secondary outcome measures were a determination of interrater reliability calculated by using Cohens *κ*. Subgroup analyses included elderly patients (≥ 65 years old) and patients with preexisting cognitive impairment (preoperative Mini-Cog-Results < 3).

## Results

**Fig. 2:**
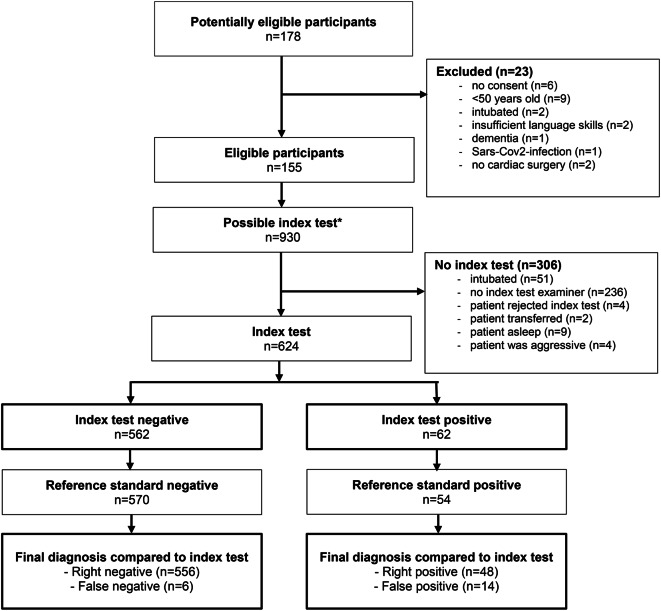
Participant flowchart

Among 178 scheduled cardiac surgery patients, 155 proved eligible between November 2021 and February 2022, 23 of which were excluded. In these, 624 paired examinations were completed on the first three postoperative days (see flowchart in Fig. 2). Aggressive patients could not be accurately examined due to lack of cooperation and no plausible disorientation assessment.

Baseline characteristics of the study population are shown in Table 1. The median age of the study population was 68 (IQR: 61–73) years. 33 (21.3%) females were included. Overall, we found an incidence of POD according to the reference rater of 9%.

**Table 1:** Patient demographics and characteristics

	All (*n* = 155)
Age (years), median (IQR)	68 (61–73)
Sex	
Male, n (%)	122 (78.7)
Female, n (%)	33 (21.3)
Weight (kg), median (IQR)	87 (75–98)
Height (cm), median (IQR)	177(172–183)
BMI (kg/m^2^), median (IQR)	27.3 (24.7–31.3)
Laboratory parameter, median (IQR)	
Leukocytes (10^9^/L)	7.49 (6.65–9.12)
Hemoglobin (g/dL)	13.9 (12.5–15.1)
Platelets (10^9^/L)	242 (195–280)
Quick (%)	104 (94–110)
Creatinine (mg/dL)	0.95 (0.83–1.15)
AST (U/L)	22 (19–28)
ALT (U/L)	22 (14–31)
Bilirubin (mg/dL)	0.5 (0.3–0.7)
CRP (mg/L)	2.8 (1.1–10.7)
ChE (kU/L)	7.7 (6.5–8.7)
Admission Comorbidities^⊕^, n (%)	
Myocardial infarction	52 (33.5)
Cerebrovascular disease	15 (9.7)
Chronic renal failure	5 (3.2)
Gastroduodenal ulcer	5 (3.2)
Tumor (+/- metastasis)	15 (9.7)
Heart failure	37 (23.9)
Diabetes mellitus (+/- organ damage)	49 (31.6)
Chronic pulmonary disease	13 (8.4)
Peripheral artery disease	6 (3.9)
Planned surgery, n (%)	
CABG	98 (63.2)
Aortic valve replacement	29 (18.7)
CABG and aortic valve replacement	18 (11.6)
Ascending aorta replacement	1 (0.6)
Other	13 (8.4)
Pre-Operative cognition baseline, median (IQR)	
Mini-Cog-Result ^±^	4 (2–5)
Orientation^‡^	5 (5–5)
RASS^°^	0 (0–0)

IQR = Interquartile range. BMI = Body mass index. AST = Aspartate-Aminotransferase. ALT = Alanine-Aminotransferase. CRP = C-Reactive Protein. ChE = Cholinesterase. CABG = Coronary Artery Bypass Graft. RASS = Richmond Agitation Sedation Scale

^⊕^ Multiple selections were possible

^°^ RASS ranges from − 5 (deeply sedated) to + 4 (aggressive)

^±^ Mini-Cog-Results are ranging from 0 to 5, a result < 3 indicates dementia

^‡^ For orientation, 1 point for each correct answer in the following categories: age, birthday, situation, location and time

**Fig. 3:**
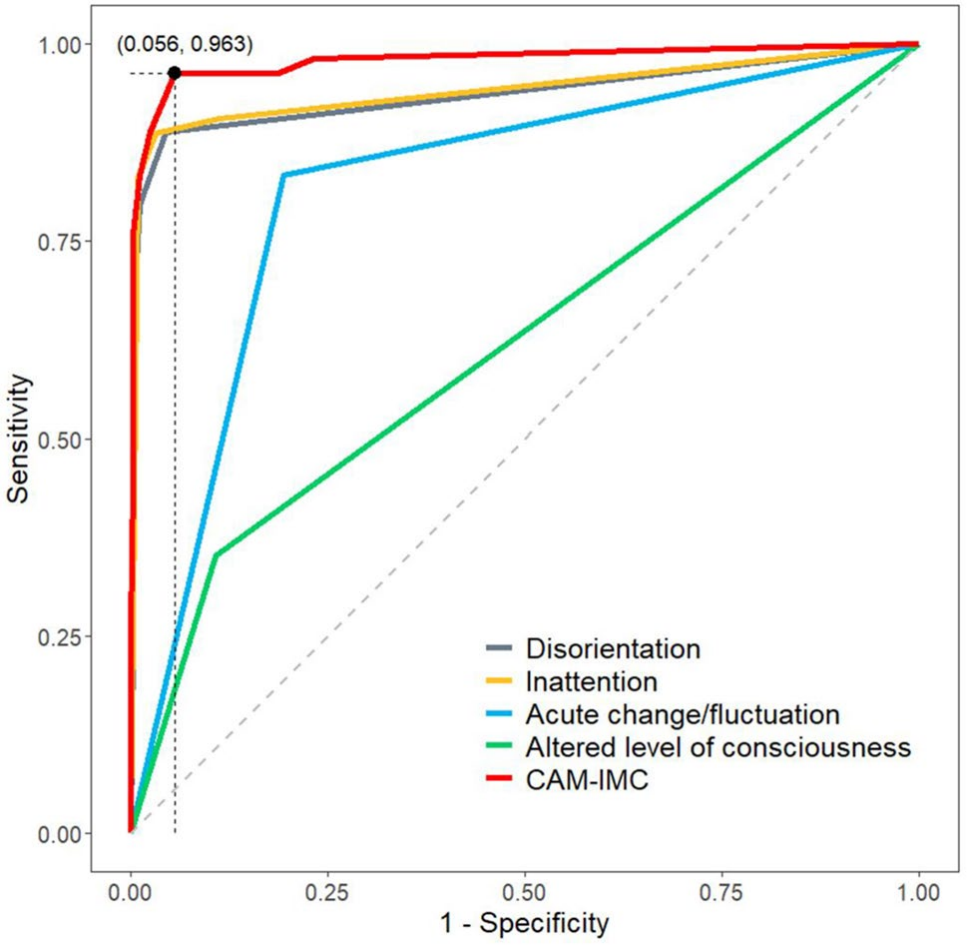
Receiver-Operating-Characteristic (ROC) curves for CAM-IMC and its items for diagnosis of postoperative delirium (POD) in non-intubated cardiac surgery patients, with the optimal cutoff (three points) marked by the Youden index

Overall, the CAM-IMC showed an AUROC of 0.98 (CI 95%: 0.96-1.00). The optimal cut-off based on the Youden-Index was chosen at three points for the CAM-IMC to determine the presence of POD. This is displayed in the ROC-analysis in Fig. 3.

Patients scoring three points or higher were considered as positive for POD, while those less than three points were considered negative.

Using the CAM-IMC with a cut-off at three points, 538 (86.2%) of 570 out of non-deliriant observations were classified correctly, whereas 32 (5.1%) observations tested false positive. Fifty-two (8.3%) of 54 of the POD positive observations were tested correctly. Two observations (0.3%) were misclassified as a false negative. Test performance values and AUROC of the CAM-IMC for different cut-off points are displayed in Table 2. Sensitivity and specificity for CAM-IMC with a cut-off at three points are 0.96 (CI 95%: 0.87-1.00) and 0.94 (CI 95%: 0.92–0.96), respectively. It performs with an AUROC of 0.95 (0.93–0.98).

**Table 2:** Diagnostic test performance of the CAM-IMC for diagnosis of postoperative delirium (POD) with different cut-off points across all patients, those with cognitive impairment, and patients aged ≥ 65 years

	CAM-IMC Score	Sensitivity (95% CI)	Specificity (95% CI)	Positive	Negative	Positive	Negative	AUROC (95% CI)
				Predictive Value (95% CI)		Likelihood-Ratio (95% CI)		
**All patients** (*n* = 624)	3	0.96 (0.87-1.00)	0.94 (0.92–0.96)	0.62 (0.51–0.72)	1.00 (0.90-1.00)	17.15 (12.20-24.11)	0.04 (0.01–0.15)	0.95 (0.93–0.98)
	4	0.89 (0.77–0.96)	0.98 (0.96–0.99)	0.77 (0.65–0.87)	0.99 (0.98-1.00)	36.19 (21.39–61.23)	0.11 (0.05–0.24)	0.93 (0.89–0.97)
	5	0.83 (0.71-0.9075)	0.99 (0.97-1.00)	0.87 (0.74–0.94)	0.98 (0.97–0.99)	67.86 (32.19-143.06)	0.17 (0.09–0.31)	0.91 (0.86–0.96)
**Cognitive impairment** (*n* = 133)	3	1.00 (0.85-1.00)	0.93 (0.86–0.97)	0.73 (0.54–0.88)	1.00 (0.96-1.00)	13.87 (7.12–27.05)	0.00 (0.00-NaN)	0.96 (0.94–0.99)
	4	0.91 (0.71–0.99)	0.97 (0.92–0.99)	0.87 (0.66–0.97)	0.98 (0.94-1.00)	33.64 (10.93–103.50)	0.09 (0.02–0.35)	0.94 (0.88-1.00)
	5	0.91 (0.71–0.99)	1.00 (0.97-1.00)	1.00 (0.83-1.00)	0.98 (0.94-1.00)	Inf (NaNInf)	0.09 (0.02–0.34)	0.95 (0.89-1.00)
**Age ≥ ** **65 years** (*n* = 398)	3	0.96 (0.86–0.99)	0.91 (0.88–0.94)	0.61 (0.49–0.72)	0.99 (0.98-1.00)	11.18 (7.90-15.82)	0.05 (0.01–0.18)	0.94 (0.90–0.97)
	4	0.92 (0.80–0.98)	0.96 (0.94–0.98)	0.77 (0.64–0.87)	0.99 (0.97-1.00)	24.68 (14.38–42.36)	0.09 (0.03–0.22)	0.94 (0.90–0.98)
	5	0.90 (0.77–0.97)	0.99 (0.97-1.00)	0.86 (0.73–0.94)	0.99 (0.97-1.00)	44.79 (21.38–93.85)	0.11 (0.05–0.24)	0.94 (0.89–0.98)

Abbreviations: n = number of examinations. CAM-IMC = Confusion-Assessment-Method for Intermediate Care. AUROC = Area Under the Receiver Operating Characteristic Curve. CI = Confidence Interval. NaN=’Not a Number’. Inf=’infinity’

CI for Sensitivity, Specificity, Positive/Negative Predictive Value Positive/Negative Likelihood-Radio was calculated using Clopper-Pearson-Method; CI for AUROC was calculated using DeLong-Method

In comparison, Sensitivity and specificity of disorientation are 0.80 (CI 95% 0.66–0.89) and 0.99 (CI 95% 0.97–0.99), respectively. Disorientation performs with an AUROC of 0.89 (CI 95% 0.84–0.95).

AUROC of inattention is 0.91 (CI 95%: 0.86–0.96), acute change or fluctuation is 0.82 (CI 95%: 0.77–0.87) and altered level of consciousness is 0.62 (CI 95%: 0.56–0.69). A comparison between the ROC of the CAM-IMC and the individual items included in the CAM-IMC is depicted in Fig. 3. The test performance values and AUROC of each individual item are depicted in Table 3.

**Table 3:** Diagnostic test performance of each CAM-IMC item for diagnosis of postoperative delirium (POD) compared to reference standard, analyzed in full cohort, cognitively impaired patients, and those aged ≥ 65 years

	Sensitivity	Specificity	Positive	Negative	Positive	Negative	AUROC (95% CI)
	(95% CI)		Predictive Value (95% CI)		Likelihood-Ratio (95% CI)		
**Whole cohort (n = 624)**							
Acute change/fluctuation	0.83 (0.71–0.92)	0.81 (0.77–0.84)	0.29 (0.22–0.37)	0.98 (0.96–0.99)	4.28 (3.49–5.25)	0.21 (0.11–0.38)	0.82 (0.77–0.87)
Altered level of consciousness	0.35 (0.23–0.49)	0.89 (0.86–0.92)	0.23 (0.15–0.34)	0.94 (0.91–0.95)	3.23 (2.10–4.98)	0.73 (0.60–0.89)	0.62 (0.56–0.69)
Inattention*	0.83 (0.70–0.92)	0.99 (0.98-1.00)	0.90 (0.78–0.97)	0.98 (0.97–0.99)	94.64 (39.21-228.43)	0.17 (0.09–0.31)	0.91 (0.86–0.96)
Disorientation	0.80 (0.66–0.89)	0.99 (0.97–0.99)	0.86 (0.73–0.94)	0.98 (0.97–0.99)	64.84 (30.68-137.06)	0.21 (0.12–0.35)	0.89 (0.84–0.95)
**Cognitive impairment (n = 133)**							
Acute change/fluctuation	0.82 (0.60–0.95)	0.76 (0.67–0.83)	0.40 (0.26–0.56)	0.95 (0.89–0.99)	3.36 (2.29–4.93)	0.24 (0.10–0.59)	0.79 (0.70–0.88)
Altered level of consciousness	0.23 (0.08–0.45)	0.91 (0.84–0.96)	0.33 (0.12–0.62)	0.86 (0.78–0.91)	2.52 (0.96–6.66)	0.85 (0.67–1.07)	0.57 (0.48–0.66)
Inattention	0.86 (0.65–0.97)	1.00 (0.97-1.00)	1.00 (0.82-1.00)	0.97 (0.93–0.99)	Inf (NaN-Inf)	0.14 (0.05–0.39)	0.93 (0.86-1.00)
Disorientation	0.95 (0.77-1.00)	0.98 (0.94-1.00)	0.91 (0.72–0.99)	0.99 (0.95-1.00)	52.98 (13.38-209.82)	0.05 (0.01–0.31)	0.97 (0.92-1.00)
**Age ≥ 65 years (n = 398)**							
Acute change/fluctuation	0.85 (0.72–0.94)	0.77 (0.72–0.81)	0.34 (0.25–0.43)	0.97 (0.95–0.99)	3.69 (2.95–4.62)	0.19 (0.10–0.38)	0.81 (0.76–0.87)
Altered level of consciousness	0.38 (0.24–0.53)	0.87 (0.83–0.90)	0.29 (0.18–0.41)	0.91 (0.87–0.94)	2.92 (1.85–4.60)	0.72 (0.57–0.90)	0.62 (0.55–0.69)
Inattention*	0.81 (0.67–0.91)	0.99 (0.97-1.00)	0.88 (0.75–0.96)	0.97 (0.95–0.99)	56.60 (23.44-136.62)	0.19 (0.11–0.35)	0.90 (0.84–0.95)
Disorientation	0.88 (0.75–0.95)	0.98 (0.96–0.99)	0.86 (0.73–0.94)	0.98 (0.96–0.99)	43.75 (20.85–91.80)	0.13 (0.06–0.27)	0.95 (0.93–0.98)

Abbreviations: n = number of examinations. CAM-IMC = Confusion-Assessment-Method for Intermediate Care. AUROC = Area Under the Receiver Operating Characteristic Curve. CI = Confidence Interval. NaN = ’Not a Number’. Inf = ’infinity’

CI for Sensitivity, Specificity, Positive/Negative Predictive Value Positive/Negative Likelihood-Radio was calculated using Clopper-Pearson-Method; CI for AUROC was calculated using DeLong-Method

Cut-Off for ANANASBAUM-test was three or more errors. Cut-Off for Disorientation was two or more errors. RASS ≠ 0 and disorientation are binary

* One Patient with missing sub-examination for this item

Two hundred eighteen paired assessments by the two index-testers showed an interrater reliability of *κ* = 0.80 (CI 95%: 0.69–0.91) in the secondary outcome. The subgroup analysis for elderly patients (≥ 65 years) and patients with cognitive impairment suggesting suspected dementia showed similar test performance values and AUROC (see Table 2). Test performance values and AUROC of each individual item are listed likewise in Table 3.

## Discussion

This cohort study evaluates the test performance of the newly developed CAM-IMC and its disorientation component in a prospective cohort study. The CAM-IMC shows a sensitivity of 0.96 and specificity of 0.94 in diagnosis of POD in non-intubated postoperative cardiac surgery patients. Its test performance is comparable to the widely used CAM-ICU demonstrating a high level of accuracy [19]. While testing for disorientation alone shows good test performance values, it does not surpass the CAM-IMC. The study indicates that using the CAM-IMC by combing the features of the CAM-ICU with assessing of disorientation as a verbal testing variable, improves effectiveness and provides excellent sensitivity and specificity for diagnosing POD. These findings highlight the importance of incorporating verbal testing for disorientation whenever patients are accessible to oral evaluation to improve POD diagnostics. The CAM-IMC outperforms each individual feature of the CAM-IMC.

By creating the CAM-IMC flowchart design as a point-based scoring system, the importance of disorientation as a central feature of POD has been reinforced. The features disorientation and inattention can each trigger a positive test result. The system assigns up to five points for disorientation and three points for inattention, thereby increasing the weight of these single features in the overall diagnosis. The categories of acute or fluctuating change and RASS≠0 have been reduced in significance and each contribute one point to the total score but are not mandatory for a positive test outcome. This is reflected in their limited ability to discriminate POD, as shown by a distinct lower AUROC compared to disorientation, inattention and overall CAM-IMC. As a result, disorientation and inattention exert the greatest influence on CAM-IMC test-results. In comparison, the CAM-ICU does not diagnose POD if “Acute change or fluctuating course of the mental status” is assessed as negative, even when inattention or disorganized thinking” is present. By employing a point-based scoring system, the CAM-IMC avoids this limitation.

The robustness of the CAM-IMC is demonstrated by the fact that index examiners with varying levels of experience, including physicians, medical students, and nursing staff were able to achieve an interrater reliability of 0.80. This underscores the ability of the CAM-IMC to provide consistent and reliable test results regardless of the experience level of the examiner. Overall, the CAM-IMC is robust across user experience levels, feasible, and will be an important assessment tool in future clinical practice. Compared with the interrater reliability of the CAM-ICU, the latter had a kappa value of κ=0.96 in the original study [19].

Strengths of this study are the thorough assessment of preoperative cognitive functions and baseline status involving the Mini-Cog and all CAM-IMC features including tests of inattention and disorientation. According to Morandi et al., it is critical to test for pre-existing neurocognitive disorders and establish a baseline cognitive status to accurately detect fluctuations or changes [31]. Our study was designed to address this concern and allowed for the detection of fluctuating changes from preoperative cognitive baseline.

The CAM-IMC achieves similar test performance values compared to the well-established 4AT and Intensive-Care-Delirium-Screening-Checklist (ICDSC). In the original validation studies, the 4AT and the ICDSC achieved a sensitivity of 89.7% and 99%, while specificity was 84.1% and 64%, respectively [21, 32]. These results were confirmed in meta-analysis, which showed a sensitivity of 0.88 for the 4AT and 0.83 for the ICDSC, and a specificity of 0.88 and 0.87 in the 4AT and ICDSC, respectively [33, 34].

Interestingly, there are notable differences in terms of disorientation testing between the CAM-IMC and the 4AT, as well as the ICDSC. The CAM-IMC assesses disorientation in five categories, including situational awareness. Both the 4AT and the ICDSC do not assess situational awareness. In contrast, the 4AT tests disorientation in four categories (age, date of birth, place, year) [21]. The ICDSC, on the other hand, tests for orientation in three categories (time, place, person) [32]. The inclusion of a situational awareness component in the CAM-IMC in disorientation testing could provide valuable information and potentially contribute to the accuracy in detecting POD. As early as 1956, Levin pointed out that disorientation, particularly in time and place, is a key indicator of delirium [1]. Accordingly, we hypothesize that testing for disorientation is crucial in diagnosing POD, as some patients may only show disorientation during POD, which was shown in a prior study by Guenther et al. [24]. These patients might not be identified by the CAM-ICU. Therefore, disorientation in three categories could trigger a positive test result.

### Limitation

The prevalence of POD in this study cohort was at the lower end of what would be expected based on the literature review [35, 36]. We found a prevalence of 9%, whereas a meta-analysis from 2021 found a prevalence range from 4.9 to 54.9% for POD in cardiac surgery patients [35]. According to a review by Stollings et al., the prevalence of POD in ICU-patients is approximately 25% [36]. Although the preoperative exclusion criterion of age below 50 years was selected to increase the prevalence of POD, as age is a known predisposing factor for delirium, the prevalence remained relatively low. It is possible that preoperative cognitive testing may have trained participants and had a pre-rehabilatory effect on cognitive status in the sense, that cognitive testing and the entire process of informing and consenting is a cognitive intervention per se. This may have contributed to a reduced occurrence of POD as shown in earlier studies [37, 38]. Additionally, delirium was monitored for only three postoperative days. Consensus-based guidelines recommend screening until the fifth postoperative day, meaning some cases of POD may have been missed [39]. The study was also conducted in a hospital with well-established delirium monitoring, education, and prevention measures, which may have led to heightened awareness and potentially lower incidence of POD. The study itself may have lowered prevalence, as the ICU staff were highly motivated to detect and prevent POD during the period of the study.

The selection criteria also limit the generalizability of the CAM-IMC to younger patients and those undergoing emergency surgery, as well as to other surgical populations. Only cardiac surgery patients were enrolled, representing a relatively homogenous cohort with typically higher incidence of POD.

Another limitation was the lack of a standardized timepoint for the assessment of POD. Evaluation was conducted alongside morning ward rounds. A maximum of 60 min were between the raters. While this approach reflects more every-day practice, it limits the comparability of standardized time points and may have resulted in missed delirious episodes due to the fluctuating nature of POD. Furthermore, patients with POD could have been missed due to low level of ICU-experience and judgement of the index raters since all levels of experience were allowed to test. Another limitation is that, while a significant portion of the index tests were completed, some were not conducted (see flowchart 1) primarily due to the unavailability of the index test raters caused by a staff shortage the COVID-19 pandemic.

## Conclusion

Recognition of POD is crucial to prevent adverse consequences for patients. Early diagnosis can help focus attention on patients with suspected POD, leading to improved care [40, 41]. The CAM-IMC provides a highly structured tool for diagnosing POD in non-intubated patients by combining the well-established CAM-ICU with ‘disorientation’. In institutions where the CAM-ICU is already established, it will be beneficial to include disorientation in the further assessment of POD in non-intubated patients. With an aging population and an increasing numbers of IMC units and non-intubated ICU patients, CAM-IMC could serve as a cornerstone in assessment of POD in such settings. It could also be used in emergency departments, post-anesthesia care units and whenever patients are at risk for delirium. Further validation and comparison of the CAM-IMC in diverse patient cohorts are needed. Future research should investigate the significance of the various features of disorientation, particularly the role and importance of situational awareness in diagnosing POD and focus on both internal and external validation of the CAM-IMC.

## Data Availability

The dataset used in the current study is available from the senior-author on reasonable request.
